# A machine learning approach to study plant functional trait divergence

**DOI:** 10.1002/aps3.11576

**Published:** 2024-04-05

**Authors:** Sambadi Majumder, Chase M. Mason

**Affiliations:** ^1^ Department of Biology University of Central Florida Orlando 32816 Florida USA; ^2^ Department of Biology University of British Columbia Okanagan Kelowna V1W5H9 British Columbia Canada; ^3^ Present address: Global Water Security Center University of Alabama 1041 Cyber Hall, Box 870206 Tuscaloosa 35487 Alabama USA

**Keywords:** ecophysiology, feature selection, gradient boosting machine, *Helianthus*, multidimensional, random forest

## Abstract

**Premise:**

Plant functional traits are often used to describe the spectra of ecological strategies used by different species. Here, we demonstrate a machine learning approach for identifying the traits that contribute most to interspecific phenotypic divergence in a multivariate trait space.

**Methods:**

Descriptive and predictive machine learning approaches were applied to trait data for the genus *Helianthus*, including random forest and gradient boosting machine classifiers and recursive feature elimination. These approaches were applied at the genus level as well as within each of the three major clades within the genus to examine the variability in the major axes of trait divergence in three independent species radiations.

**Results:**

Machine learning models were able to predict species identity from functional traits with high accuracy, and differences in functional trait importance were observed between the genus and clade levels indicating different axes of phenotypic divergence.

**Conclusions:**

Applying machine learning approaches to identify divergent traits can provide insights into the predictability or repeatability of evolution through the comparison of parallel diversifications of clades within a genus. These approaches can be implemented in a range of contexts across basic and applied plant science from interspecific divergence to intraspecific variation across time, space, and environmental conditions.

Ecophysiologists have long strived to explain the variation in ecological strategy among plant species using trait axes (Grime, 1977; Westoby, 1998; Reich et al., 2003; Wright et al., 2004; Reich, 2014), where ecological strategy is defined as the manner in which plant species sustain themselves in a specific environment (Westoby, 1998). In this regard, the use of functional traits has been central, specifically referring to those morphological, physiological, chemical, or phenological traits that indirectly contribute to evolutionary fitness through effects on growth, survival, and reproduction (Violle et al., 2007). Functional traits typically shape plant resource use and environmental interactions, and ecophysiologists have often sought to summarize interspecific variation in plant performance and functionality from only a handful of selected proxy traits that represent broader axes of trait variation (Westoby et al., 2002). Analyzing plant ecophysiology through the lens of a few traits (or more specifically, the trait axes they are thought to represent) permits researchers to make global comparisons across many hundreds to thousands of species across the global diversity of ecosystems (Díaz et al., 2016).

Examples of few‐trait paradigms of ecological strategies include the competitor–stress tolerator–ruderal triangle (CSR; Grime, 1977), the leaf–height–seed scheme (LHS; Westoby, 1998), the leaf economics spectrum (LES; Wright et al., 2004), and the plant economics spectrum (PES; Reich, 2014). For example, the CSR triangle posits that the relative selective pressures of competition, abiotic stress, or biomass‐destroying disturbance select for specific trait combinations in plants (Grime, 1977). These strategies are mediated by plant functional traits that either permit a rapid investment in vegetative growth and competition for resources in stable high‐resource environments; investment in dense, persistent tissues that allow the maintenance of metabolic activity in stable low‐resource environments; or investment in early reproduction and a high output of offspring in unstable environments. The LHS scheme attempts to explain variation in plant ecological strategies based on variation in three axes: leaf construction and productivity, plant stature and competitiveness for light, and the relative provisioning of propagules during reproduction (Westoby, 1998). Such a scheme permits the categorization or relative placement of species in trait space on a global scale, and each of these three axes are known to vary quite considerably between species at any level of the other two axes (Westoby, 1998). Modern efforts to convert these qualitative descriptions into quantitative axes are based on very few leaf traits (Pierce et al., 2013). The LES (Wright et al., 2004) was developed with the aim of identifying a single worldwide axis of leaf ecophysiological variation, based on the relative investments of carbon and nutrients during leaf construction, leaf productivity per unit time, and realized leaf lifespan. This axis was thought to reflect the leaf‐level contribution to whole‐plant ecological strategies ranging from fast growth and low tolerance to resource‐related stressors, to slow growth and high tolerance to resource‐related stressors. This idea has been further expanded into a stem economics spectrum (e.g., Baraloto et al., 2010), a root economics spectrum (e.g., Mommer and Weemstra, 2012), a flower economics spectrum (e.g., Roddy et al., 2020), and indeed a holistic whole PES (Reich, 2014), which integrates these organ‐level axes given how resources flow among the organs of the plants. All of these spectra generally seek to capture trait variation in relation to carbon, nutrient, and water resources and how such trait variation can explain growth and fitness across plant species in biomes around the world. These trait‐based spectra of ecological strategies can be used to address how trait variation impacts species distributions, community assembly processes, and ecosystem‐scale functions.

While these spectra have typically been initially investigated and defined using collections of species with large interspecific trait variation, they tend to be created using small sets of traits selected based on some a priori expectation of trait importance from the existing body of knowledge about plant physiology, as well as the relative ease of measuring a given trait in a reproducible way on many hundreds or thousands of plants. Indeed, researchers have actively focused on generating lists of traits that can be measured easily and suggested that an approximate consensus be sought for a ranked list of “important” traits that reflect ecological strategies (Westoby et al., 2002). If such an approximate consensus regarding universally “important” traits could be found, it would immensely help researchers to compare ecophysiological studies in different systems, conduct meta‐analyses across studies, and forecast future vegetation dynamics under a changing climate (Westoby, 1998).

As a lineage of species diversifies across environmental gradients, natural selection acts on various functional traits given their role in resource acquisition and use and, by definition, their indirect impact on plant fitness through effects on growth, survival, and reproduction (Caruso et al., 2020). However, while natural selection arising from resource availability, competition for resources, or disturbance is certainly very important, these are not the only sources of selection that shape plant populations. Both natural and sexual selection arise from pollinators and other mutualists, herbivores and other natural enemies, and non‐resource abiotic factors such as ultraviolet radiation, thermal regimes, soil texture, or many other aspects of the abiotic and biotic environment (Geber and Griffen, 2003; Caruso et al., 2019). Indeed, a recent systematic review and meta‐analysis of all published studies employing a selection analysis of functional traits determined that morphological, chemical, physiological, and phenological traits all appear to experience a similar magnitude of directional selection in natural populations (Caruso et al., 2020).

Given the multivariate natures of the environment and whole‐plant phenotypes, the small number of core functional traits used to define the CSR, LHS, or PES paradigms may or may not have been particularly important during the evolutionary history of phenotypic divergence during the diversification of a given lineage. This means that trait‐first approaches, where a small number of traits such as plant height, specific leaf area, or seed size are assessed in a study system because they are deemed ecologically “important” at a global interspecific scale, have the potential to miss traits that are more important aspects of phenotypic divergence during the diversification of lineages evolving under lineage‐specific evolutionary constraints and contingency (Donovan et al., 2011; Blount et al., 2018). As a number of studies have pointed out, individual plant lineages or specific functional groups of species occupying a portion of the larger global variation may or may not share pairwise trait relationships observed across larger interspecific data sets (e.g., Edwards et al., 2014; Klimešová et al., 2015; Mason and Donovan, 2015; Niinemets, 2015; Anderegg et al., 2018). Independently identifying the most “important” functional traits with broad phenotypic diversification within a study system thus has strong utility, as it permits a test of whether these existing paradigmatic trait spectra are actually the predominant axes of interspecific trait variation for a given lineage, or whether other plant traits demonstrate far stronger divergence among focal species and warrant further investigation for their functional role in the adaptation and contribution to ecological strategies. The phenotypic traits that most strongly separate species in high‐dimensional multivariate trait space are hereafter referred to in this work as the strongly divergent traits, and we here demonstrate an analytical approach for identifying these traits in multi‐species multivariate trait data sets.

To accomplish this, we examine trait patterns within the genus *Helianthus* L. (sunflower) and in each of three distinct clades within the genus (the annual clade, the southeastern perennial clade, and the large perennial clade) that reflect independent radiations across habitats. *Helianthus* species are abundant across North America (Heiser et al., 1969) and can be found in a wide variety of habitats spanning biomes that include arid, semi‐arid, subtropical, and temperate regions, and edaphic conditions from rock outcrops to sand dunes to wetlands. Along with diversity of habitat, a concomitant broad variation in traits is also observed, making *Helianthus* an excellent model for studying functional trait divergence. Members of the annual clade have short lifespans ranging from true annuals that live only three months to facultative perennials that may live for a few years, and all members reproduce exclusively through seeds. Members of the large perennial clade are rhizomatous erect perennials with some lifespans exceeding a decade, all of which are deciduous, dying back to rhizomes each year, and with varying degrees of vegetative reproduction by clonal spread in addition to seed production. The southeastern perennial clade contains a combination of deciduous rhizomatous erect perennials and quasi‐evergreen basal rosette perennials, all with long lifespans, which reproduce using a mix of seed production and clonal reproduction through rhizomes or root crown buds.

These three clades are distinct in their life histories and growth forms, but each contains considerable overlapping diversity in morphological, physiological, chemical, and phenological functional traits among species, and each has diversified across largely parallel environmental gradients (Mason and Donovan, 2015; Mason et al., 2016, 2017a, 2017b). This work aims to assess whether the same traits are strongly divergent within each of the three clades given the contingency of diversification from the three distinct common ancestors, which speaks to how repeatable or predictable the evolution of functional traits is—an outstanding question in the evolution of plant functional traits (Caruso et al., 2020). Given that many major functional trait paradigms (e.g., CSR, LHS, PES) make predictions that parallel shifts in trait values should be observed under adaptation to the same environmental pressures or adaptation to the same ecological strategy largely regardless of the plant lineage in question (e.g., the evolution of higher leaf mass per area in drier and less fertile environments), the approach employed here provides a means to examine the similarity of multivariate trait divergences across lineages and gauge the applicability of global paradigms to a given study system.

Our analytical approach uses machine learning (ML)–based descriptive and predictive modeling techniques to objectively identify the traits that are strongly divergent among species relative to within‐species variation. In the context of ML, descriptive models are used to explain data and gain insights, whereas predictive models are used to make forecasts (van Klompenburg et al., 2020). In the application of these approaches for our purposes, the descriptive modeling approach facilitates the ranking of plant traits according to their relative “importance” in relation to species divergence in a multivariate trait space, as well as identifying a handful of optimal subsets of traits relevant to species delineation. The predictive modeling approach validates these findings by identifying species from their traits. This methodology is somewhat analogous to the non‐ML method of using variance partitioning to investigate the magnitude of interspecific vs. intraspecific variation in functional traits across ecological data sets (Albert et al., 2010; Kazakou et al., 2014; Prieto et al., 2017); however, these latter approaches are almost always univariate and beholden to a range of assumptions (linearity, bivariate normality, and homoscedasticity), in contrast to the multivariate ML‐based approach we demonstrate here. Multivariate ML‐based approaches permit the modeling of species trait divergences in a manner analogous to the ways in which multivariate suites of traits evolve in nature, including non‐linear and strongly non‐bivariate‐normal relationships among traits. The feasibility of using interpretable ML‐based models in the trait‐based classification of species and the identification of relevant traits in this context has been demonstrated previously using interpretable ML classifiers such as decision trees (Almeida et al., 2020). Such methods of identification have many parallels with traditional dichotomous keys, which are widely used as a convenient and inexpensive method of species identification (Tilling, 1984). Applying tree‐based ML algorithms to the identification of plant species provides a method that does not rely heavily on subjective a priori researcher determinations of which traits are the most informative. Analogously, we here leverage multivariate ML‐based approaches to identify sets of functional traits that are strongly divergent among species and therefore strong candidates for traits of evolutionary and ecological significance worthy of confirmatory assessment for adaptive and functional importance.

## METHODS

### Data sources and plant materials

Functional trait data from four separate publications (Mason and Donovan, 2015; Mason et al., 2016, 2017a, 2017b) were acquired from the Dryad Digital Repository and aggregated into a single data set. The relevant trait data came from the same common garden experiment comprising 28 diploid wild *Helianthus* species grown under high‐resource greenhouse conditions (Mason and Donovan, 2015). Each species was represented by 2–4 unique seed accessions derived from populations across the range of each species, with approximately 5–8 individual plants (biological replicates) per population. The 28 species represent over 80% of all diploid nonhybrid species within the genus *Helianthus*, distributed across the diploid backbone of the genus phylogeny (Timme et al., 2007). The combined data set used in this study (Table S1, see Supporting Information) included leaf morphological, ecophysiological, and defensive chemistry traits; whole‐plant growth, biomass allocation, and phenology traits; and floral morphological and ecophysiological traits all measured on individual replicate plants (rows in our combined data set), such that measured trait values are paired at the individual plant level rather than at population or species levels. While further trait data on root and stem ecophysiology exist for these species from separate experiments (Bowsher et al., 2016), such data are not paired at the individual plant level and thus cannot be included in our present analysis.

### Statistical software and packages

Data cleaning workflows and ML pipelines were designed using packages written in the R programming language (version 4.2.1; R Core Team, 2022). The relevant code was executed on a standard laptop with 8 GB of RAM to ensure the wide accessibility of the approach. All the code needed to reproduce the analyses in this work is provided on GitHub: https://github.com/SamMajumder/MachineLearningFunctionalTraitDivergence.

### Data cleaning and data preparation

**Figure 1:**
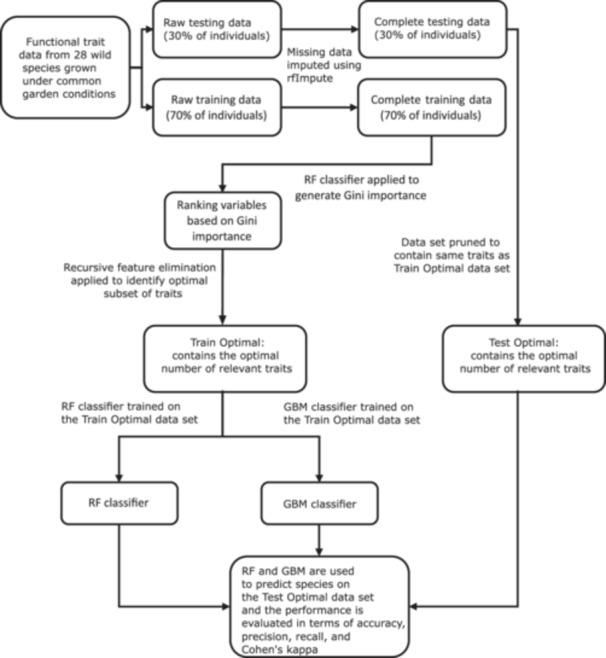
Complete workflow of the entire analysis procedure. The data were divided into training (70% of individuals) and test (30% of individuals) data sets by random sampling. Missing data were imputed using a random forest (RF) algorithm using the R function *rfImpute* from the package *randomForest*. A RF classifier was applied to the imputed training data and the traits were ranked based on Gini importance. A recursive feature elimination method was implemented using the imputed training data, and the optimal subset of ecologically relevant traits for species diversification was identified. The traits that were not in the optimal subset were excluded from both the training (Train Optimal) and the test (Test Optimal) data sets. Two predictive models were trained on this reduced training data set. One predictive model was built using the RF classifier, while the other was built using the gradient boosting machine (GBM). These two models were validated using the test data set and compared with each other using metrics such as overall accuracy, precision, recall, and the F1 score.

The steps undertaken to clean and prepare the data, as well as subsequent analysis steps, are outlined in Figure 1. The full names of the traits were changed to an abbreviated format for ease of manipulation and use within the analysis pipeline; a full list of traits and their corresponding abbreviations are provided in Table S2. This was achieved using packages within the R tidyverse (Wickham et al., 2019). About 15% of the data were missing from the data set, as computed and visualized in Figure S1. The training data set was created using 70% of the individuals selected at random (Table S3), with the remaining 30% used as the test data set (Table S4). Missing data were imputed using a proximity matrix from a random forest (Breiman, 2003), which was implemented using the package *randomForest* (Liaw and Wiener, 2002). The training and test data set were imputed separately to avoid data leakage. Genus‐level questions were addressed by considering the entire training data set during the analysis, while clade‐level questions were addressed by dividing the larger training data set into three parts representing three major monophyletic clades within the genus: the large perennial clade, the annual clade, and the southeastern perennial clade sensu Stephens et al. (2015). Species not contained within these three clades were excluded from clade‐level analyses. The genus‐level training and test data sets exhibited a slight class imbalance, for example, *H. annuus* L., *H. radula* (Pursh) Torr. & A. Gray, and *H. silphioides* Nutt. had twice as much representation in the whole data set (approx. *n* = 48) than the other species (approx. *n* = 24) given their use as phytometers within the original experiment, and had smaller variances due to differences in the number of replicate plants and/or missing trait data. These small imbalances existed in the clade‐level data sets as well.

### Applying ML to rank, select, and evaluate the ecological relevance of functional traits to interspecific divergence

In this study, we employ two tree‐based ensemble ML classification algorithms, namely random forest (RF) and gradient boosting machine (GBM), in various ways. Random forest is an ensemble ML algorithm that builds several decision trees, and each tree is trained on a bootstrapped version of the original data set, where the data are randomly sampled with replacement (Pal, 2005; Valletta et al., 2017). The predictions of each decision tree are then averaged across all trees, and this process in conjunction with bootstrapping the data set is called “bootstrap aggregating” (Valletta et al., 2017). The data left out of the bootstrapping procedure during the training process are called the “out of bag” data (OOB) and are used to optimize the classifier (Cutler et al., 2012; Valletta et al., 2017). The GBM is another ensemble tree‐based algorithm where each tree in the ensemble predicts the error of the previous decision tree and each subsequent tree attempts to reduce this error, thereby sequentially improving the prediction accuracy (Friedman, 2001). This powerful ML algorithm is highly flexible and customizable for a wide array of data types and ML applications (Natekin and Knoll, 2013). The GBM contains four hyperparameters, which can be tuned for the purposes of controlling the complexity of the predictive model (Friedman, 2001; Zhou et al., 2019; Ridgeway, 2024). These hyperparameters describe the number of decision trees built by the ensemble, the minimum number of observations for each tip of the decision tree (each species), and the learning rate and convergence across decision trees (Zhou et al., 2019).

By utilizing RF and RF‐based frameworks, it was possible to rank the functional traits based on their relative importance, identify the optimal subset of relevant traits, and isolate the core set of strongly divergent traits. First, the importance of all traits in the data set was computed using Gini impurity within a RF framework (Breiman, 2001, 2003). The use of Gini impurity in the feature selection stage involves the quantification of the quality of splits in a tree‐based classifier like RF during the decision‐making process, and this leads to the creation of efficient trees that in turn contribute to the improvement of the predictive task. At each node of a classification tree, selecting a feature or variable for branching is a crucial decision for the purposes of building a classification tree via training (Laber and Murtinho, 2019) and thus, within its framework, a method to compute feature importance is necessary (Pal, 2005). An attribute that creates the best separation between the classes during the creation of a split in the tree will contribute to the highest decrease in the Gini impurity value and would thus have the highest importance among the list of attributes (Nembrini et al., 2018). This method of computing variable importance is called the mean decrease of impurity (MDI), and allows for ranking the traits based on their importance and facilitates the development of preliminary insights in relation to ecological relevance of the traits. We implemented RF in this work by using the R package *randomForest* (Liaw and Wiener, 2002).

Recursive feature elimination (RFE) was used to identify the strongly divergent traits (Guyon et al., 2002), resulting in the reduction of the data set to contain only these traits. This selection was achieved using an objective methodology (RFE) rather than a subjective one based on researcher opinion. Recursive feature elimination is a backward feature elimination technique whereby, through a recursive process, the most important features (here the optimal subset of plant traits) are selected by building multiple models with the training data. A ranking system keeps track of the overall importance of each feature. With each iteration, the feature with the lowest rank is eliminated. In RFE, a ML classifier is used to build multiple models; the choice of classifier is determined by the user. In this work, the ML classifier chosen was a RF, and the method used to determine the importance of features and to rank and eliminate plant trait variables within the framework of the RFE was the mean decrease of accuracy (MDA) method, proposed alongside Gini impurity (Breiman, 2001). In this method, a baseline prediction accuracy is calculated using the OOB data. The value of a variable is then permuted, and this causes a change in the prediction accuracy, which is then recorded. The difference between the accuracies is averaged across all decision trees in the RF ensemble and is normalized by standard error of the differences. The importance of the variable in question is the decrease in accuracy seen after permuting its original value. These steps are then repeated for all variables and their corresponding importance is recorded. To compare the findings from RF (variable importance computed through MDI) and RFE (variable importance computed through MDA) to more traditional methods examining interspecific vs. intraspecific variation in plant functional traits, a linear mixed model was implemented in the *lme4* package (Bates et al., 2015) to perform variance partitioning among individuals within populations, among populations within species, and among species.

The presence of correlated variables can be an impediment when attempting to identify the most relevant predictors in a data set. In our data set, due to the presence of many correlated functional traits, we implemented RF within RFE during the feature selection process. This implementation is referred to in literature as the RF‐RFE framework and is designed to combat correlations between predictors in the data set when selecting for the best predictors (Gregorutti et al., 2017; Darst et al., 2018). By recursively eliminating a small number of features per loop, RF‐RFE attempts to eliminate dependencies in the data set and collinearity that may exist within the model. This has been exhibited in simulation experiments (Gregorutti et al., 2017) and in real‐world data sets (Darst et al., 2018). We implemented RFE in this work using the *caret* package (Kuhn, 2008). The plant traits within the optimal subset were retained in the training data and the rest were discarded.

Two predictive classifiers, one trained using RF and the other using GBM, were then trained on the training data and their predictive capabilities were evaluated by applying them to the test data. This step was incorporated into our analysis to evaluate whether the traits deemed evolutionarily relevant were due to overfitting. The traits identified as important by an overfit model might not be relevant in relation to the outcome, which may lead to inaccurate future predictions (Smith, 2018) as well as to erroneous conclusions regarding the manner in which the variables relate to the outcome in the biological system. To quantify the predictive capabilities of RF and GBM using the strongly divergent traits as predictors, we used the overall classification accuracy alongside class‐based metrics including precision, recall, and F1 score, calculated on the test data set. Classification accuracy refers to the proportion (or percentage) of individuals correctly predicted by the model, recall refers to the proportion of actual positives correctly identified, while precision refers to the proportion of correct positive identifications. Put simply, precision is defined as the ratio of true positives to the sum of true positives and false positives, while recall is defined as the ratio of true positives to the sum of true positives and false negatives. Specifically, in this study, the overall accuracy refers to the capability of the classifier to correctly predict any given species in the data set, while recall conveys how many of an individual species were correctly predicted. Precision is the measure of the quality of the prediction of individual species, while the F1 score is the harmonic mean of precision and recall. It combines the information from precision and recall into a single number, thus facilitating our understanding of the predictive performance of our models at a species‐specific level. We also used Cohen's kappa (Cohen, 1960), scaled between 0 and 1, to assess the agreement between the predictions made by the classifiers and actual labels of the species, considering the agreement that could occur by chance. A kappa value close to 1 signifies a high level of agreement between the classifier's predictions and the actual labels, indicating the strong performance and reliability of the classifier.

An interactive dashboard (available at https://sammajumder.shinyapps.io/TraitDivergenceSunflowers/) was created, which highlights the trait ranking and the divergent traits at both the genus and clade level. It also showcases trait divergence patterns among the strongly divergent traits at both the genus and the clade level. The dashboard contains interactive plots, which were created using the R packages *ggplot2* (version 3.4.2; Wickham, 2016) and *plotly* (Sievert, 2020), while the dashboard framework was created using the *shiny* package (Chang et al., 2022).

## RESULTS

### Importance ranking of traits at the genus and clade levels

**Figure 2:**
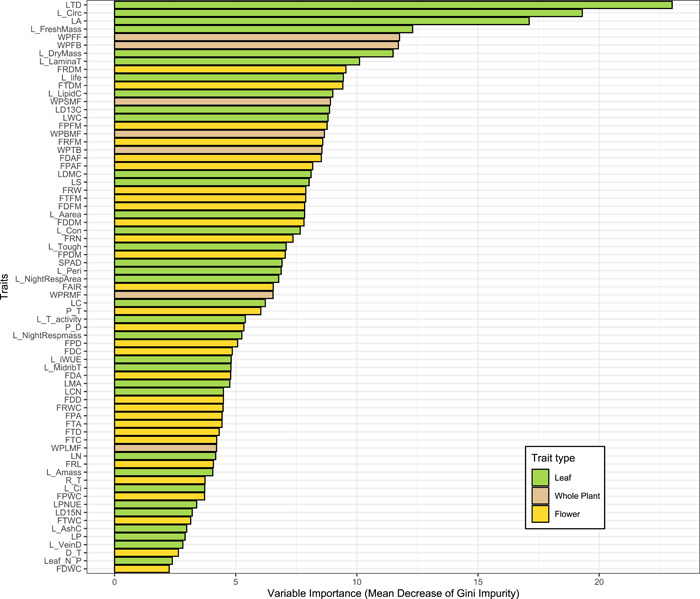
Relative importance of all 71 traits at the genus level, computed using Gini impurity by applying a random forest classifier to the training data. This was used to rank all the traits in the data set. Complete unabbreviated trait names are presented in Table S1 and should be used as a reference when viewing the relative importance of the traits presented.

On the scale of relative importance computed using the mean decrease of impurity (Figure S2), traits such as leaf trichome density, leaf size and shape, lamina thickness, and whole‐plant reproductive phenology were among the most important traits for accurate species classification in the genus‐level model (Figure 2, Table S10). Among the least important traits were those related to leaf nutrient chemistry and gas exchange, and floral morphology and water content. For each of the three clade‐level models, many of the same traits were important for accurate species classification, but with some differences among clades. In the large perennial clade model, the most important traits included leaf size and shape, leaf water use efficiency, whole‐plant reproductive phenology, and floral size, while the least important traits were leaf gas exchange and floral morphology (Table S12, Figure S10). In the annual clade model, the most important traits included leaf trichome density, leaf size, floral size, whole‐plant reproductive phenology, and plant size, while the least important traits were leaf nutrient chemistry, gas exchange, and water content (Table S14, Figure S8). In the southeastern perennial clade model, the most important traits included leaf size and shape, leaf trichome density, leaf solidity, whole‐plant reproductive phenology, and plant size, while the least important traits were leaf gas exchange, leaf lifespan, leaf mass per area, and leaf toughness (Table S16, Figure S12).

Comparing several commonly assessed plant functional traits (those of the LES and LHS scheme) between genus and clade level, we observe differences in trait variable rankings that inform patterns of trait divergence at different evolutionary scales. Leaf area exhibited a consistently high ranking at both the genus level and within each of the three clades, indicating that leaf size is a major component of interspecific multivariate trait divergence in both recent and deeper timescales. Leaf lifespan ranked much higher at the genus level (Figure 2, Table S10) than within clades, indicating that leaf lifespan had a higher degree of importance in the divergence of the common ancestors of the three clades in the multivariate trait space and relatively less importance for the subsequent interspecific divergence within each clade. This likely reflects the divergence between the common ancestors of the annual and large perennial clades (typically short leaf lifespans) and the common ancestor of the southeastern perennial clade (typically long leaf lifespans). The area‐based photosynthetic rate had a low ranking across the genus and southeastern perennial clades (Table S16, Figure S12), but a higher rank in the large perennial (Table S12, Figure S10) and annual (Table S14, Figure S8) clades, suggesting a stronger role played by photosynthetic rate in the overall interspecific phenotypic variation within those clades. Conversely, total plant biomass, predictive of stature under the LHS scheme, ranked very high in the annual clade compared with the large perennial and southeastern perennial clades, as well as at the genus level, where they ranked lower. Other traits related to the LES or LHS schemes, such as leaf mass per area and leaf nitrogen content, were found to rank low to moderate at both the genus level and within the three clades. Overall, while some traits contained within existing functional trait paradigms were found to be highly predictive of species identity and therefore interspecific phenotypic divergence, other traits within these paradigms were found to be of low importance for capturing multivariate trait divergence among species.

### Optimal subset of relevant traits identified using RFE

**Figure 3:**
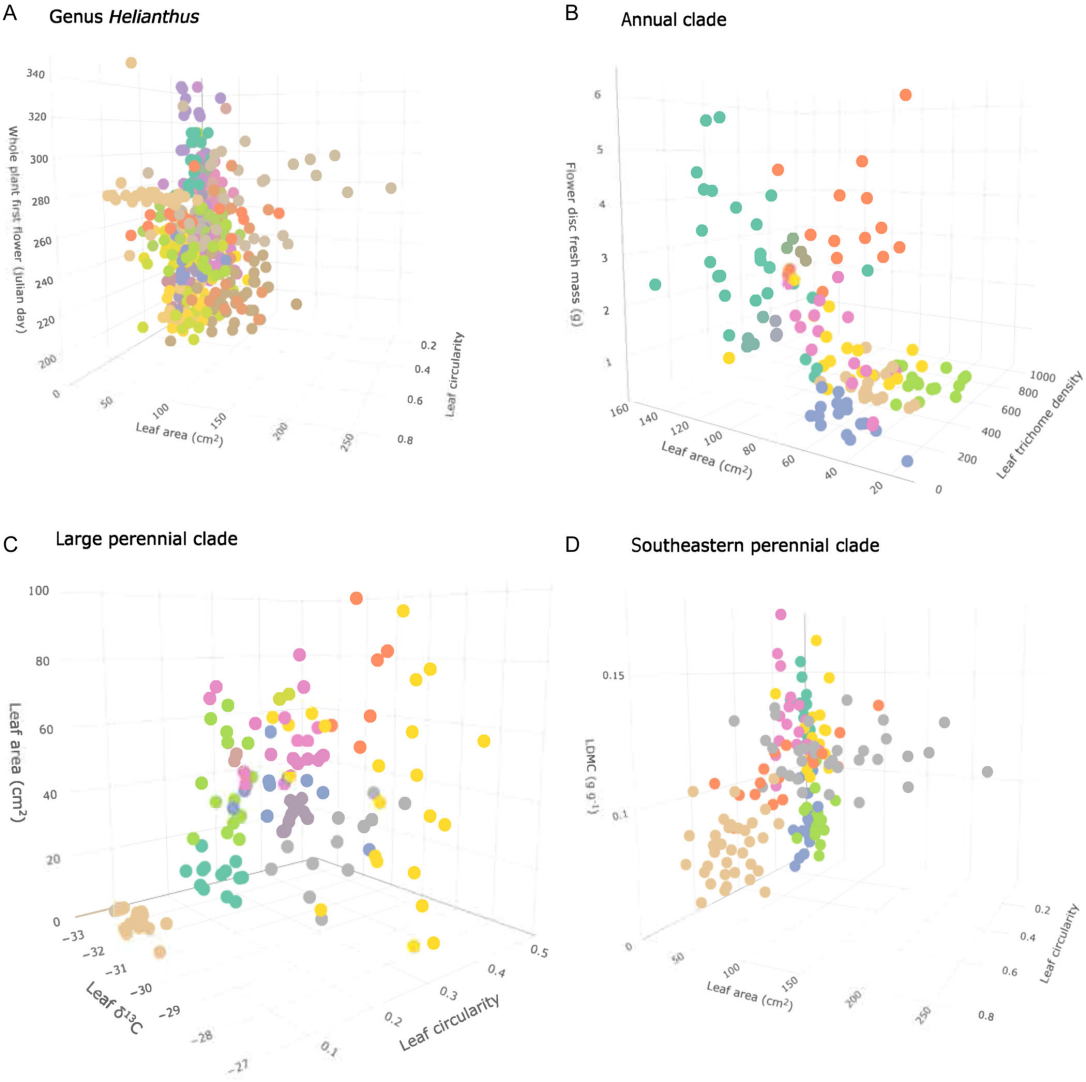
Species divergence along three strongly divergent trait axes at the genus and the three clade levels. (A) Distribution of individual plants in the trait space described by leaf circularity, leaf area, and the first flower in 28 different species within the genus *Helianthus*. (B) Distribution of individual plants in the trait space described by leaf trichome density, leaf area, and flower disk fresh mass in the species within the annual clade. (C) Distribution of individual plants in the trait space described by leaf area, leaf circularity, and leaf carbon isotope ratio in the species within the large perennial clade. (D) Distribution of individual plants in the trait space described by leaf circularity, leaf area, and leaf dry matter content (LDMC) in species within the southeastern perennial clade.

The core set of ecophysiological traits that strongly delineate species in a multivariate trait space were identified using RFE, and are referred to as strongly divergent traits. At the genus level, leaf traits such as size (leaf area, leaf mass), shape (leaf circularity), and leaf trichome density were identified as strongly divergent traits alongside whole‐plant reproductive phenology (first bud, first flower) (Table S11, Figure S6). At the clade level, several of the strongly divergent traits identified aligned with those identified at the genus level; for example, leaf size (leaf area, leaf mass) and total plant biomass were strongly divergent within all three clades, while leaf trichome density was highly divergent within the annual (Table S15, Figure S8) and southeastern perennial clades (Table S17, Figure S12). In addition, reproductive phenology (first bud, first flower) was strongly divergent within both the large perennial (Table S13, Figure S10) and southeastern perennial clades (Table S17, Figure S12). The divergence of species across three strongly divergent trait axes at the genus and clade levels is exhibited in Figure 3A (genus), 3B (annual), 3C (large perennial), and 3D (southeastern perennial).

### Comparison of univariate and multivariate results

When comparing the results of our non‐linear multivariate approach to traditional univariate variance partitioning, we find some similarities in the results at both the genus and clade levels. Most of the traits that exhibited a high relative importance computed through the mean decrease of impurity (ranking of traits) and that are deemed to be strongly divergent by RFE at the genus level using our approach also exhibited high among‐species variation relative to the among‐population and among‐individual levels (Table S6, Figure S2). This pattern is also seen in the perennial (Table S7, Figure S3), annual (Table S8, Figure S4), and southeastern perennial clades (Table S9, Figure S5), as traits with high interspecific variation considered in a univariate approach would be expected to be captured by our multivariate approach as strongly divergent among species.

There are, however, some important differences between the two approaches; for example, while floral morphology traits such as flower disk diameter or petal area fraction exhibited high among‐species variation for the genus (Table S6, Figure S2) and for the southeastern perennial clade (Table S9, Figure S5), these traits had low relative variable importance via the mean decrease of impurity at the genus (Figure 2, Table S10), perennial (Table S12), and southeastern perennial levels (Table S16). The same low relative importance via the mean decrease of impurity was also true for leaf ecophysiological traits such as water content, which exhibited high variation at the genus (Table S6, Figure S2), perennial (Table S7, Figure S3), and southeastern perennial clades (Table S9, Figure S4). Indeed, many traits that exhibited high among‐species variation (>40%), including many floral morphology traits, were excluded from the optimal subset by RFE for the annual clade, as were leaf nutrient chemistry and gas exchange traits (Table S5).

### Classification models

To validate the RFE findings, we evaluated our classification models on the test data set using metrics such as overall accuracy, precision, recall, and the F1 score (Figure 1). At the genus and the clade levels, RF performed roughly equally to GBM in correctly classifying the species from trait data, as evidenced by the metrics computed on the unseen test data set. The overall accuracy at the genus level was 95.7% for RF (precision: 0.93, recall: 0.91, F1 score: 0.91) and 91.3% for GBM (precision: 0.84, recall: 0.83, F1 score: 0.82); for the annual clade was 92.1% for RF (precision: 0.87, recall: 0.86, F1 score: 0.85) and 90.8% for GBM (precision: 0.84, recall: 0.84, F1 score: 0.83); for the perennial clade was 96.0% for RF (precision: 0.95, recall: 0.93, F1 score: 0.93) and 93.8% for GBM (precision: 0.90, recall: 0.89, F1 score: 0.89); and for the southeastern perennial clade was 95.9% for RF (precision: 0.94, recall: 0.93, F1 score: 0.93) and 96.3% for GBM (precision: 0.94, recall: 0.93, F1 score: 0.93).

## DISCUSSION

### What classification models can tell us about trait evolution in *Helianthus*

A range of studies have questioned the applicability of global‐scale plant functional trait paradigms to smaller evolutionary scales, finding that major axes of diversification or underlying trait–trait relationships differ from global paradigms within well‐studied diverse lineages, among species within diverse genera, or among populations within widespread species (e.g., Edwards et al., 2014; Klimešová et al., 2015; Mason and Donovan, 2015; Niinemets, 2015; Anderegg et al., 2018). Given concerns about the applicability of global trait spectra at smaller scales, the handful of plant functional traits used to represent such global spectra may have little relevance to species phenotypic diversification at these scales. The source studies used for the present work (Mason and Donovan, 2015; Mason et al., 2016, 2017a, 2017b) examined a wide array of ecophysiological, chemical, morphological, and phenological traits across the genus *Helianthus*, and typically interpreted the evolution of these traits in light of global plant functional trait paradigms. However, taking a more holistic view using ML methods across a wide range of traits, we here find relatively lower interspecific divergence in the conventionally “important” leaf ecophysiological traits, such as those of the leaf economics spectrum (gas exchange, leaf nutrients, leaf mass per area), and far higher divergences in leaf size and morphology, as well as whole‐plant phenology, size, and biomass allocation. This comports with the earlier finding that whole‐plant phenological and biomass allocation traits are more strongly evolutionarily correlated with native habitat environmental variables than are leaf economics traits (Mason and Donovan, 2015; Mason et al., 2017a). Similarly, divergences in leaf size and shape have previously been found to be evolutionarily correlated with native habitat environmental variables, as well as with integrated water‐use efficiency and other leaf‐level resource‐use traits within the genus (Mason and Donovan, 2015). Floral size traits have also previously been found to be evolutionarily correlated with native habitat environmental variables (Mason et al., 2017b). The results obtained here indicate that, in diversifying across wide gradients of water and nutrient availability, temperature, and growing season length, each of the three major clades within the genus diversified along somewhat different multivariate trait axes, perhaps constrained by limited variation in other traits in the context of growth form and life history. The ML approach implemented here permits a traits‐first examination of the species phenotypic divergences across the genus, which can be compared to existing functional trait paradigms to determine whether the traits underlying global spectra are the most important aspects of species phenotypic divergence.

For *Helianthus*, our results suggest that the LES (Wright et al., 2004) is likely not the primary axis of trait divergence within the genus, although several traits related to the LHS scheme (Westoby, 1998) are found to be among the strongly divergent traits. However, the differences observed in variable importance among clades indicates that patterns of species trait divergence under habitat diversification are only partially repeated even when arising from a recent common ancestor. The importance of divergence in leaf size and shape suggests a more important functional role for these traits in relation to adaptation across environments than is currently recognized for sunflowers (Nicotra et al., 2011). Likewise, the importance of leaf trichome density suggests that the known roles of trichomes in mediating interactions with the abiotic environment (temperature, radiation, and water; Bickford, 2016) and natural enemies (herbivores and pathogens; Levin, 1973; Dalin et al., 2008) are important under wild *Helianthus* diversification and ripe for more detailed study, particularly given that trichomes are known to produce diverse secondary metabolites within cultivated *H. annuus* (Aschenbrenner et al., 2013, 2015, 2016; Spring et al., 2015).

### General utility for assessing interspecific trait divergence

The approach presented here identifies trait axes upon which a set of species have most strongly separated in multivariate trait space. These traits are good candidates for subsequent confirmatory study of their functional importance and role in adaptation and diversification, but this method alone does not provide any information about evolutionary process or ecological significance. Importantly, the application of our approach to other groups of species will be most informative when species evolutionary relationships are well understood, and when species as biological units of organization are well supported by phylogenetic as well as geographic, ecological, cytotype, and reproductive information (e.g., for *Helianthus*; Heiser et al., 1969; Stephens et al., 2015). In situations where poorly studied taxa are defined solely by the morphological species concept or other trait characteristics without accompanying evidence of reciprocal monophyly, the application of any classification approach using taxa as categories has the risk of circularity. In such circumstances (e.g., polyphyletic species described by morphology alone), classification approaches may inflate the ranked importance of the morphological characters used to delineate taxa relative to chemical, phenological, or physiological traits.

Additionally, any approach for examining the relative importance of various plant functional traits to interspecific phenotypic divergence will be influenced by the choice of traits included. Using the present analysis as an example, we include a large number of leaf, floral, and whole‐plant traits, but were unable to include other functional traits, such as the belowground ecophysiology of fine roots and mycorrhizal collaboration (Bergmann et al., 2020; Carmona et al., 2021; Weigelt et al., 2021), carbohydrate storage and budbank traits (Klimešová et al., 2015, 2018), or detailed stem vascular transport and structural traits (Chave et al., 2009; Baraloto et al., 2010). Researchers should of course be cautious to consider the scope of traits included in any analysis (including trait class, organ system, or even developmental stage) to avoid overinterpreting the importance of traits included relative to the traits excluded.

As demonstrated for *Helianthus*, the tree‐based ML approach presented here can be trained on functional trait data from nested or parallel levels of biological organization to assess the degree of concordance in the phenotypic divergences observed, comparing independent diversifications of multiple clades or genera or families across the same environmental gradients or geographical regions to assess the degree of predictability in functional trait evolution (Caruso et al., 2020). Additionally, these methods have utility as a screening tool for identifying highly divergent traits among study species for subsequent inquiries into ecological function, as well as potentially semi‐automating the building of dichotomous keys for identification purposes in confusing or difficult groups of species.

### Other applications of ML classification models in plant science

In ecological and agricultural studies, data sets often contain continuous trait data collected at different scales. In such situations, tree‐based ML methods provide reliable and accurate predictions without the need for data scaling. The tree‐based approaches employed in this study can be applied to answer similar types of category‐assignment or category‐divergence questions in plant science. The two ensemble ML algorithms used in this study, RF and GBM, have been implemented in a wide variety of plant science applications, including identifying disease resistance proteins (Simón et al., 2022), crop yield prediction (Jeong et al., 2016), and predicting plastic phenotypic traits from genetic and environmental data (Westhues et al., 2021). While both RF and GBM are designed to handle high‐dimensional data sets, the computation time varies between the two due to the difference in the underlying training process. For RF, decision trees are built in parallel, while for GBM decision trees are built sequentially due to the nature of the iterative optimization method. Biological studies pertaining to genomic selection revealed that GBM trees are more accurate than RF trees (Ogutu et al., 2011), whereas RF seems to outperform GBM when applied to data sets containing primarily categorical variables (Cha et al., 2021). These types of approaches can be used to identify major physiological, genomic, or biochemical features contributing to differences among crop cultivars, plant developmental stages, or geographic intraspecific variation among populations. Tree‐based models trained on biological data from known categories (e.g., species, genotypes, ecotypes, ontogenetic stage, disease states) can also be used to identify category membership for unknown individuals, as well as to potentially identify closely related types (e.g., closely related species, disease states) that have not yet been described by observing the probability of prediction relative to well‐described types (e.g., species, disease states). We hope future studies in plant ecophysiology and agriculture will adopt non‐linear multidimensional methods, such as those presented in this study, alongside more traditional frequentist statistical methods, especially when the assumptions of linear models are violated for large multidimensional data sets.

## AUTHOR CONTRIBUTIONS

S.M. and C.M.M. designed the study. S.M. wrote all code and conducted all analyses. S.M. created the figures and wrote the manuscript with input from C.M.M. All authors approved the final version of the manuscript.

## Supporting information


**Figure S1**. Percentage of missing values in the entire data set, which includes the 71 functional traits along with population and species columns.
**Figure S2**. Estimated relative variation partitioned for all 71 traits at the genus level, with variance partitioned into species, population, and residual (within‐population among‐individual) components.
**Figure S3**. Estimated relative variation partitioned for all 71 traits within the large perennial clade, with variance partitioned into species, population, and residual (within‐population among‐individual) components.
**Figure S4**. Estimated relative variation partitioned for all 71 traits within the annual clade, with variance partitioned into species, population, and residual (within‐population among‐individual) components.
**Figure S5**. Estimated relative variation partitioned for all 71 traits within the southeastern perennial clade, with variance partitioned into species, population, and residual (within‐population among‐individual) components.
**Figure S6**. Optimal subset of divergence‐relevant traits at the genus level, ascertained using a recursive feature elimination (RFE) method on the data set. The variable importance was calculated using the mean decrease of accuracy from a random forest classifier within the framework of RFE.
**Figure S7**. Relative importance of all 71 traits within the annual clade, computed using Gini impurity by applying a random forest classifier to the training data. This was used to rank all traits in the data set.
**Figure S8**. Optimal subset of divergence‐relevant traits within the annual clade, ascertained using a recursive feature elimination (RFE) method on the data set. The variable importance was calculated using the mean decrease of accuracy from a random forest classifier within the framework of RFE.
**Figure S9**. Relative importance of all 71 traits within the large perennial clade, computed using Gini impurity by applying a random forest classifier to the training data. This was used to rank all the traits in the data set.
**Figure S10**. Optimal subset of divergence‐relevant traits within the large perennial clade, ascertained by using a recursive feature elimination (RFE) method on the data set. The variable importance was calculated using the mean decrease of accuracy from a random forest classifier within the framework of RFE.
**Figure S11**. Relative importance of all 71 traits within the southeastern perennial clade, computed using Gini impurity by applying a random forest classifier to the training data. This was used to rank all the traits in the data set.
**Figure S12**. Optimal subset of divergence‐relevant traits within the southeastern perennial clade, ascertained using a recursive feature elimination (RFE) method on the data set. The variable importance was calculated using mean decrease of accuracy from a random forest classifier within the framework of RFE.

**Table S1**. Functional trait data from 28 diploid wild *Helianthus* species.
**Table S2**. List of all functional traits used in this study along with their corresponding abbreviations.
**Table S3**. Training data. These data were used to perform descriptive modeling and to train machine learning classifiers.
**Table S4**. Testing data. These data were used to validate the predictive capabilities of the machine learning classifiers trained on the training data.
**Table S5**. Traits that were not part of the optimal subset of relevant traits at the genus and clade level.
**Table S6**. Variance partitioned between species, population, and corresponding residuals at the genus level.
**Table S7**. Variance partitioned between species, population, and corresponding residuals at the perennial clade level.
**Table S8**. Variance partitioned between species, population, and corresponding residuals at the annual clade level.
**Table S9**. Variance partitioned between species, population, and corresponding residuals at the southeastern perennial clade level.
**Table S10**. Relative importance of each trait at the genus level, calculated using Gini impurity by fitting a random forest model to the training data.
**Table S11**. Optimal subset of ecologically relevant traits at the genus level, identified by recursive feature elimination.
**Table S12**. Relative importance of each trait at the perennial level, calculated using Gini impurity by fitting a random forest model to the training data.
**Table S13**. Optimal subset of ecologically relevant traits at the perennial level, identified by recursive feature elimination.
**Table S14**. Relative importance of each trait at the annual level, calculated using Gini impurity by fitting a random forest model to the training data.
**Table S15**. Optimal subset of ecologically relevant traits at the annual level, identified by recursive feature elimination.
**Table S16**. Relative importance of each trait at the southeastern perennial level, calculated using Gini impurity by fitting a random forest model to the training data.
**Table S17**. Optimal subset of ecologically relevant traits at the southeastern perennial level, identified by recursive feature elimination.

## Data Availability

All data used in this study are available from the Dryad Digital Repository (https://doi.org/10.5061/dryad.110s9, https://doi.org/10.5061/dryad.5hq56, https://doi.org/10.5061/dryad.v3824, and https://doi.org/10.5061/dryad.8m0q0), and the combined data set used for modeling can be found in the Supporting Information. All code used in this analysis as well as the data can be found at https://github.com/SamMajumder/MachineLearningFunctionalTraitDivergence.
