# Supplementary material for: A machine learning approach to study plant functional trait divergence
Source: Appl Plant Sci. 2024 Apr 5;12(5):e11576. doi: 10.1002/aps3.11576 (PMC11443442; doi:10.1002/aps3.11576)
Supplement: Supplementary file 1 — Figure S1. Percentage of missing values in the entire data set, which includes the 71 functional traits along with population and species columns. Figure S2. Estimated relative variation partitioned for all 71 traits at the genus level, with variance partitioned into species, population, and residual (within‐population among‐individual) components. Figure S3. Estimated relative variation partitioned for all 71 traits within the large perennial clade, with variance partitioned into species, population, and residual (within‐population among‐individual) components. Figure S4. Estimated relative variation partitioned for all 71 traits within the annual clade, with variance partitioned into species, population, and residual (within‐population among‐individual) components. Figure S5. Estimated relative variation partitioned for all 71 traits within the southeastern perennial clade, with variance partitioned into species, population, and residual (within‐population among‐individual) components. Figure S6. Optimal subset of divergence‐relevant traits at the genus level, ascertained using a recursive feature elimination (RFE) method on the data set. The variable importance was calculated using the mean decrease of accuracy from a random forest classifier within the framework of RFE. Figure S7. Relative importance of all 71 traits within the annual clade, computed using Gini impurity by applying a random forest classifier to the training data. This was used to rank all traits in the data set. Figure S8. Optimal subset of divergence‐relevant traits within the annual clade, ascertained using a recursive feature elimination (RFE) method on the data set. The variable importance was calculated using the mean decrease of accuracy from a random forest classifier within the framework of RFE. Figure S9. Relative importance of all 71 traits within the large perennial clade, computed using Gini impurity by applying a random forest classifier to the training data. This was used to rank a [file APS3-12-e11576-s002.pdf]

Majumder and Mason-Applications in Plant Sciences 2024

DOI 10.1002/aps3.11576

Supplemental figures for "A machine learning approach to studyplant functional trait divergence"

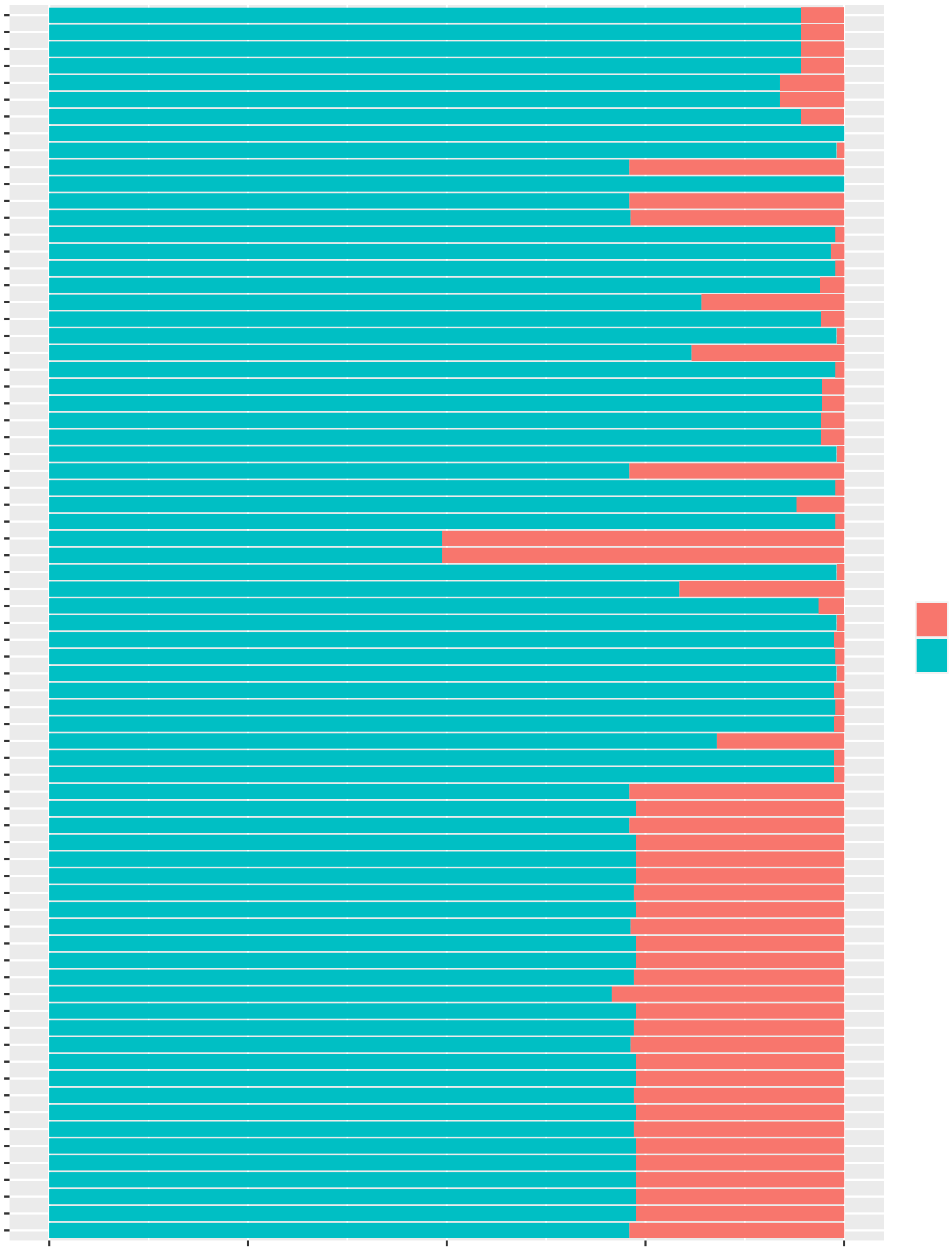

Figure S1. Percentage of missing values in the entire data set, which includes the 71 functional traits along with population and species columns.

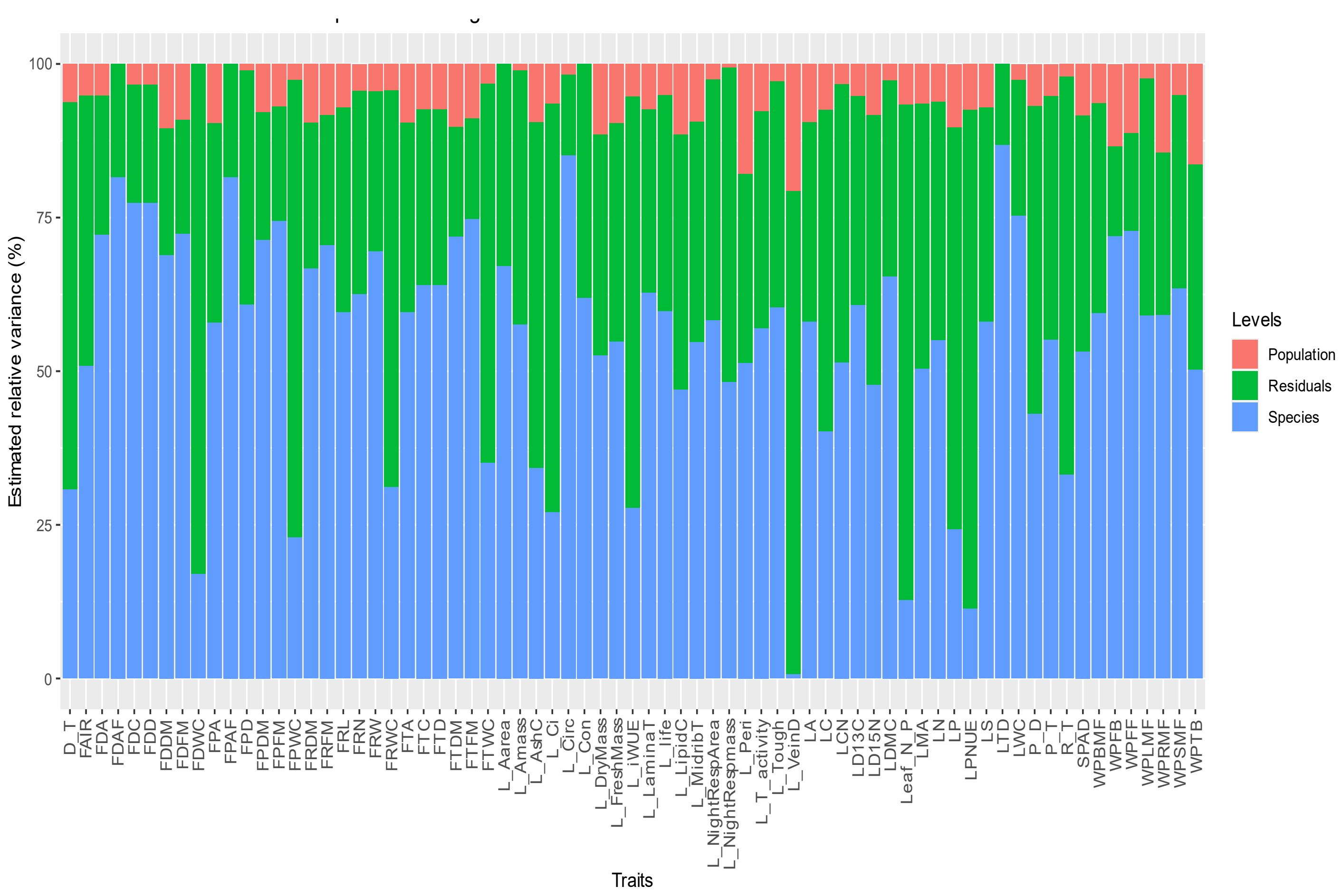

Figure S2. Estimated relative variation partitioned for all 71 traits at the genus level, with variance partitioned into species, population, and residual (within-population among-individual) components.

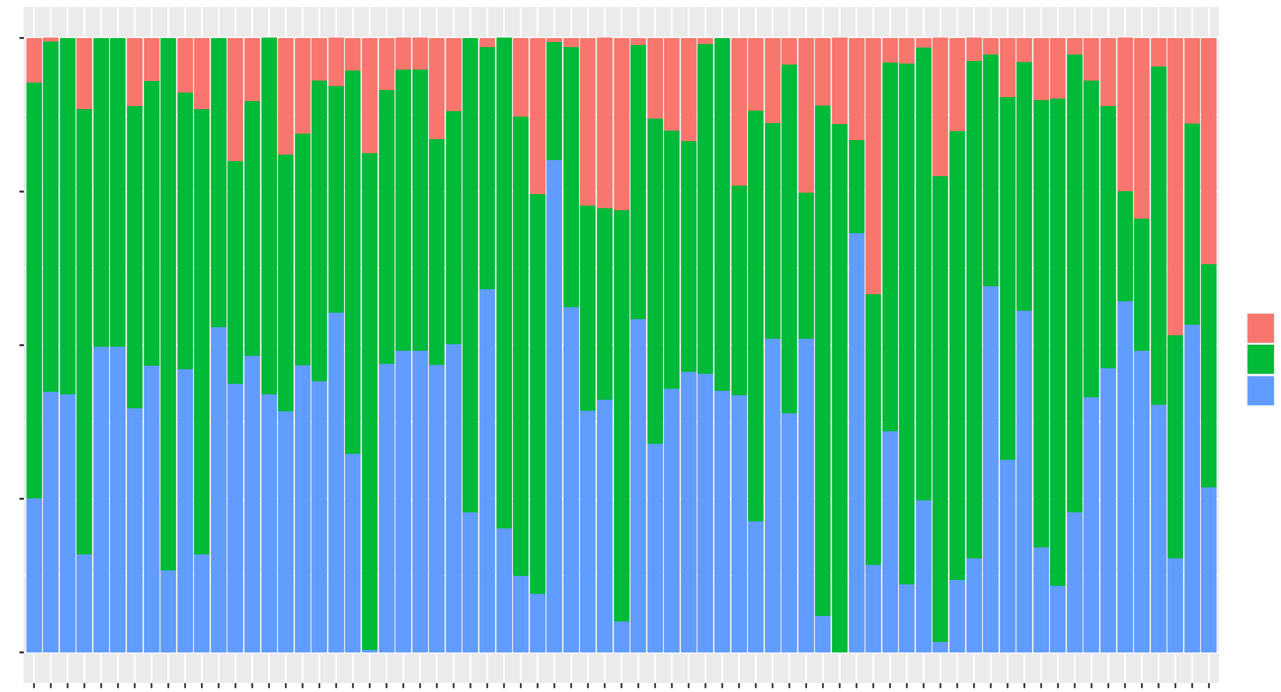

Figure S3. Estimated relative variation partitioned for all 71 traits within the large perennial clade, with variance partitioned into species, population, and residual (within-population among-individual) components.

DOI 10.1002/aps3.11576

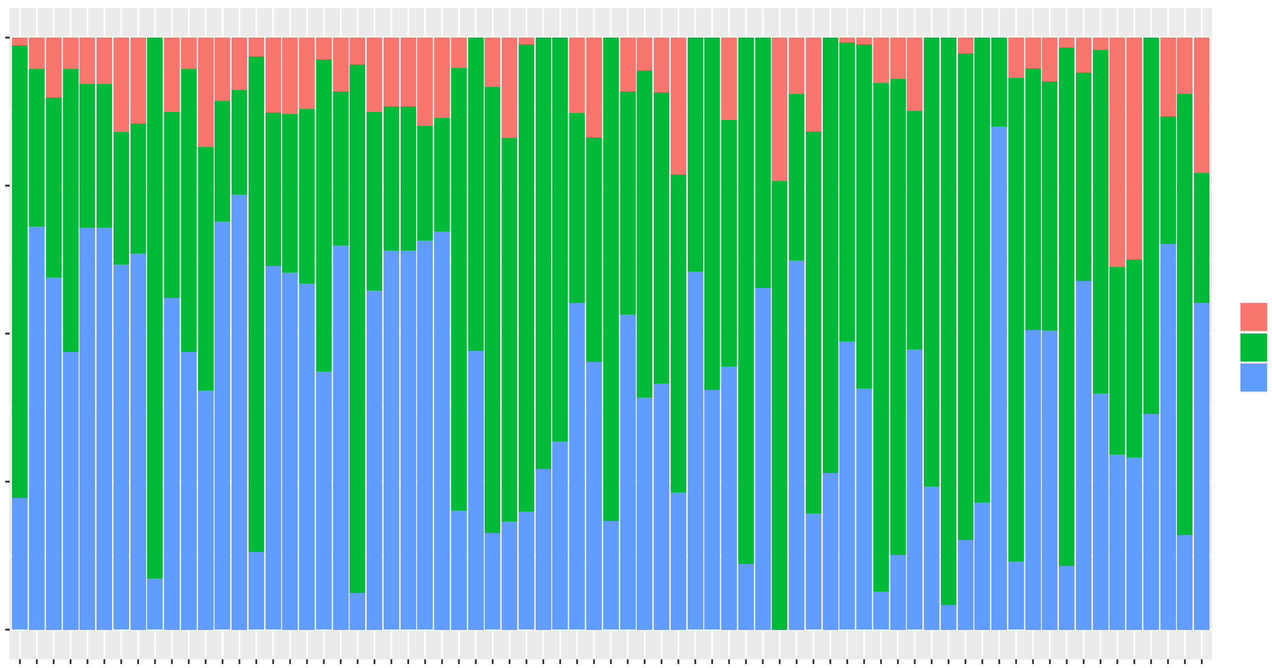

Figure S4. Estimated relative variation partitioned for all 71 traits within the annual clade, with variance partitioned into species, population, and residual (within-population among-individual) components.

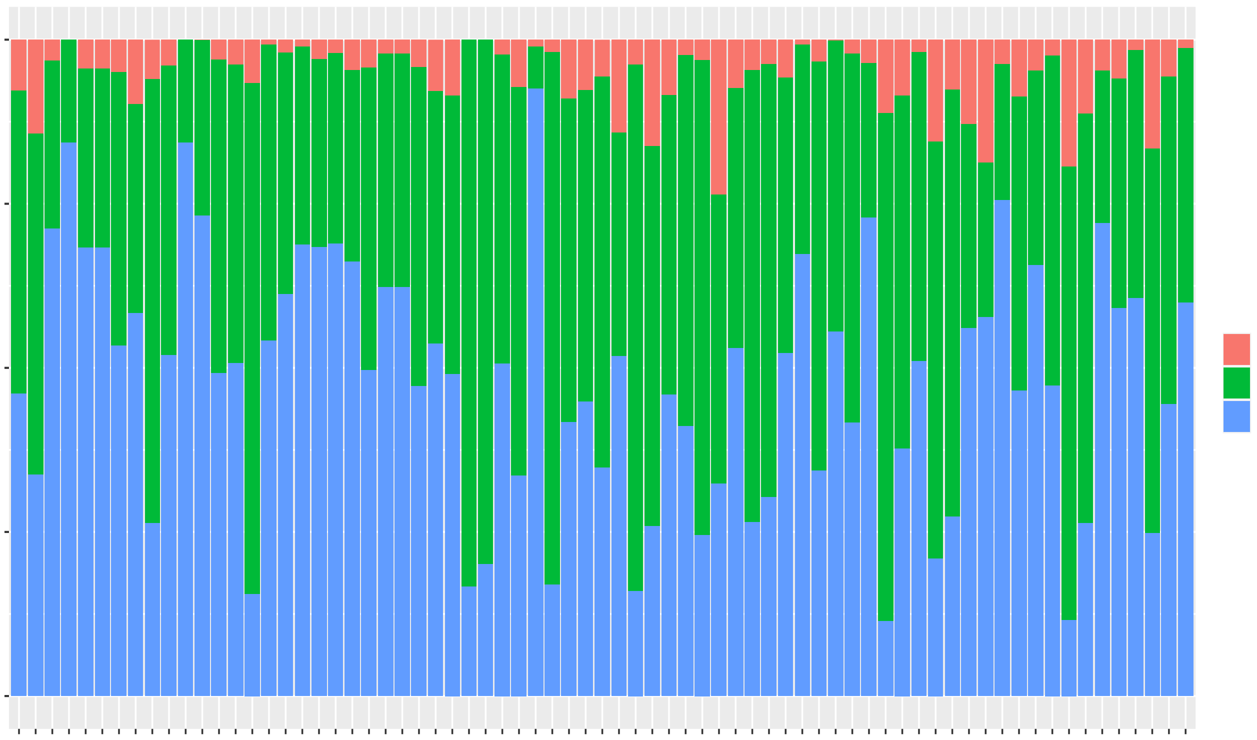

Figure S5. Estimated relative variation partitioned for all 71 traits within the southeastern perennial clade, with variance partitioned into species, population, and residual (within-population among-individual) components.

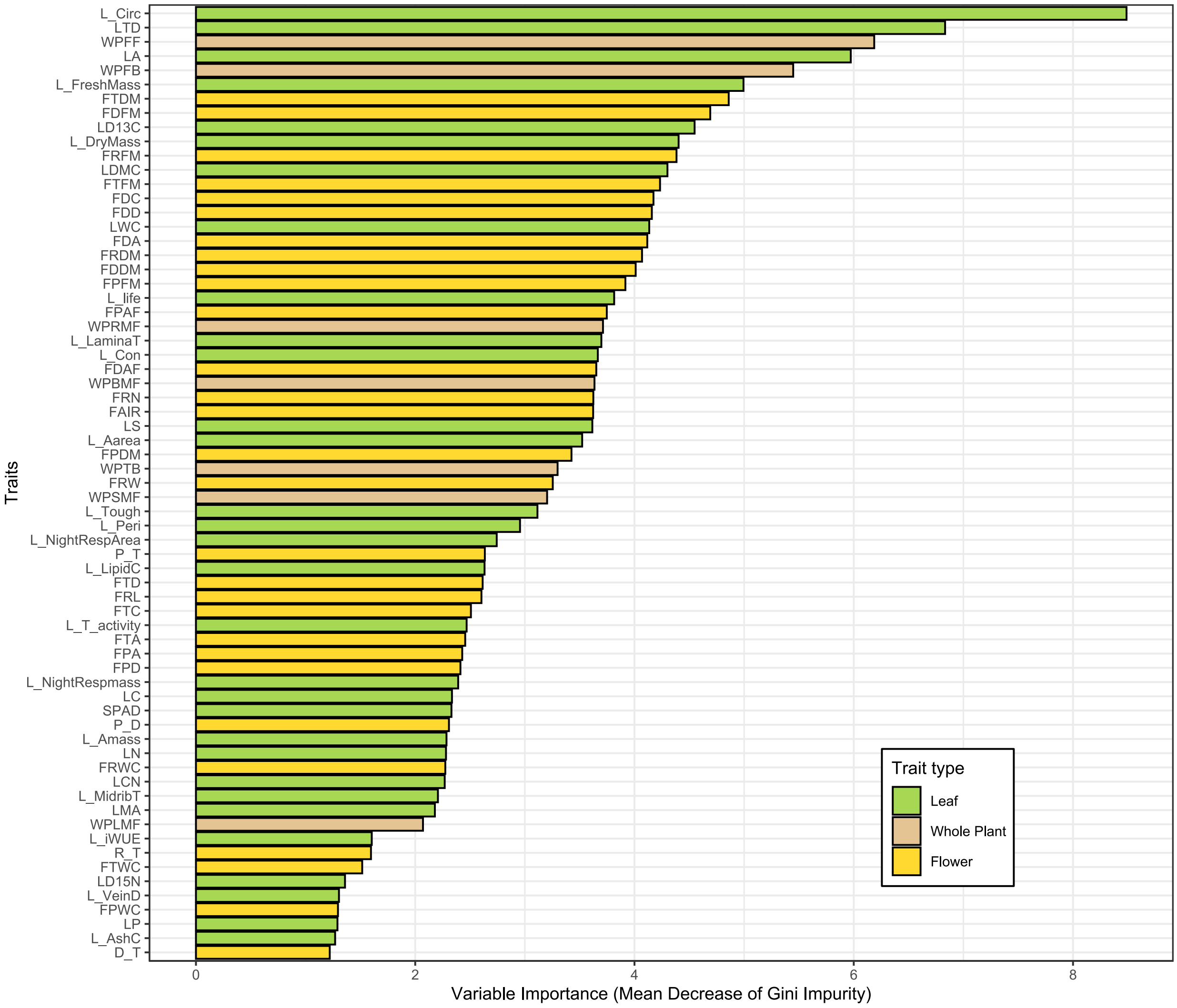

Figure S6. Optimal subset of divergence-relevant traits at the genus level, ascertained using a recursive feature elimination (RFE) method on the data set. The variable importance was calculated using mean decrease of accuracy from a random forest classifier within the framework of RFE.

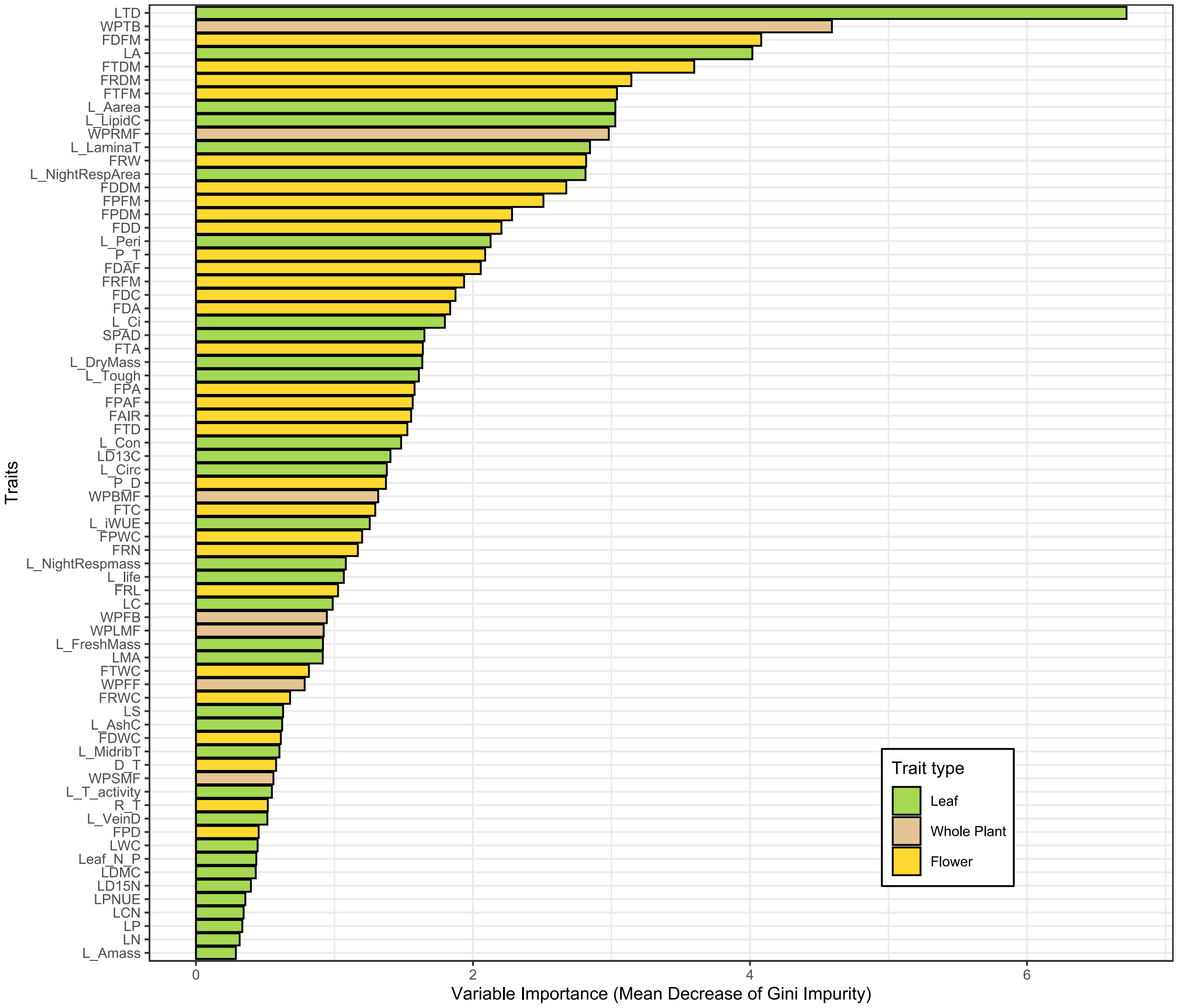

Figure S7. Relative importance of all 71 traits within the annual clade, computed using Gini impurity by applying a random forest classifier to the training data. This was used to rank all traits in the data set.

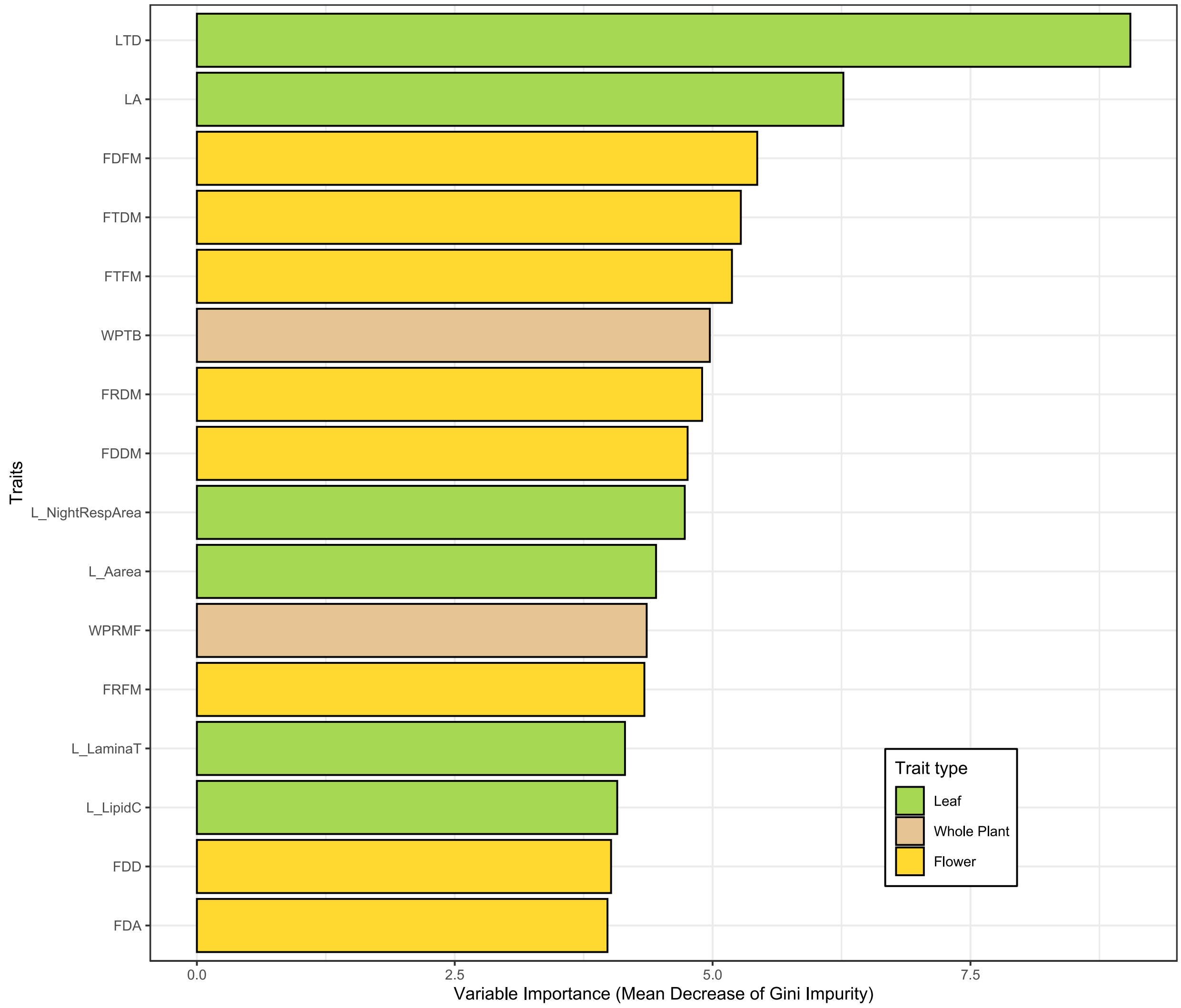

Figure S8. Optimal subset of divergence-relevant traits within the annual clade, ascertained using a recursive feature elimination (RFE) method on the data set. The variable importance was calculated using the mean decrease of accuracy from a random forest classifier within the framework of RFE.

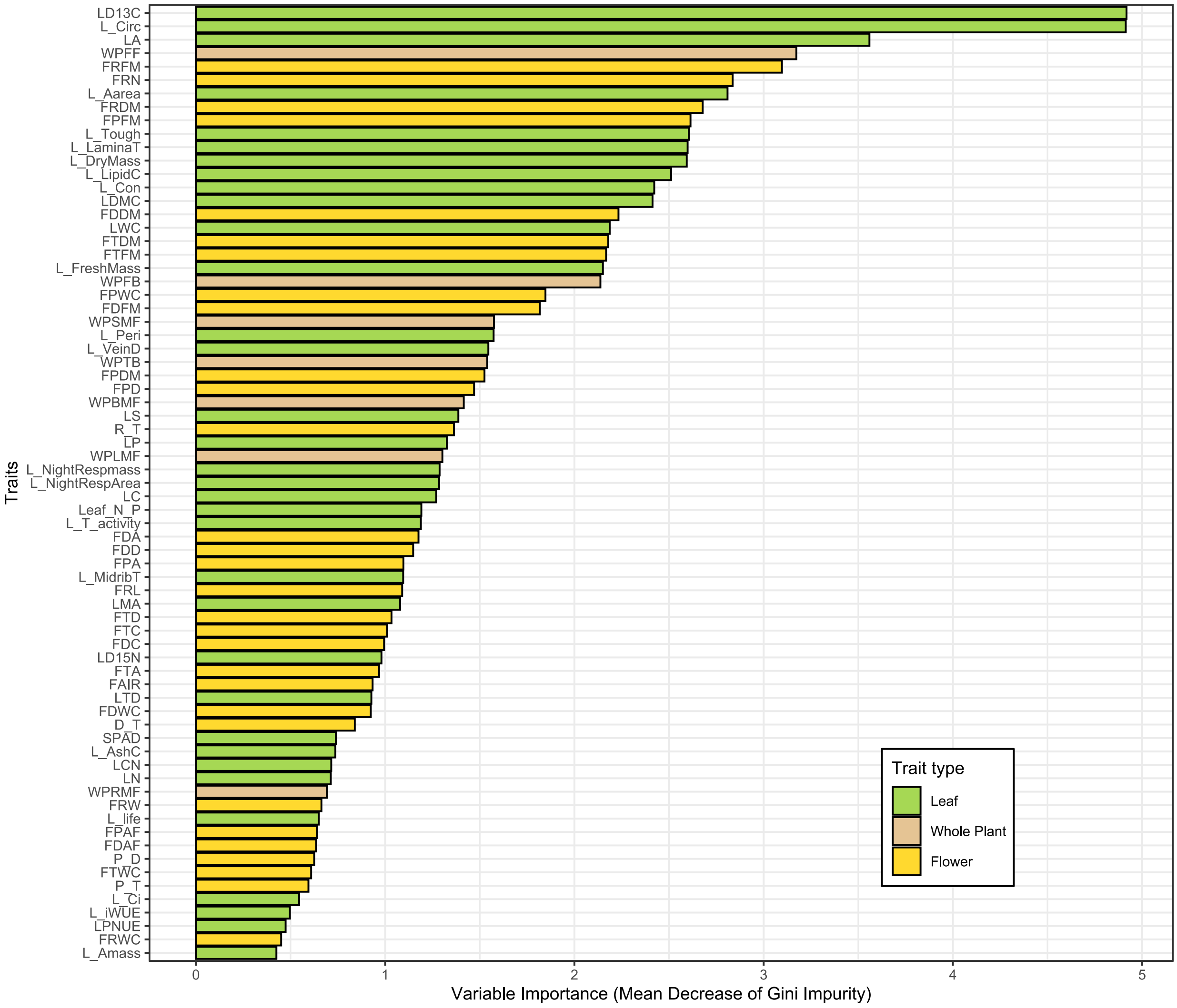

Figure S9. Relative importance of all 71 traits within the large perennial clade, computed using Gini impurity by applying a random forest classifier to the training data. This was used to rank all the traits in the data set.

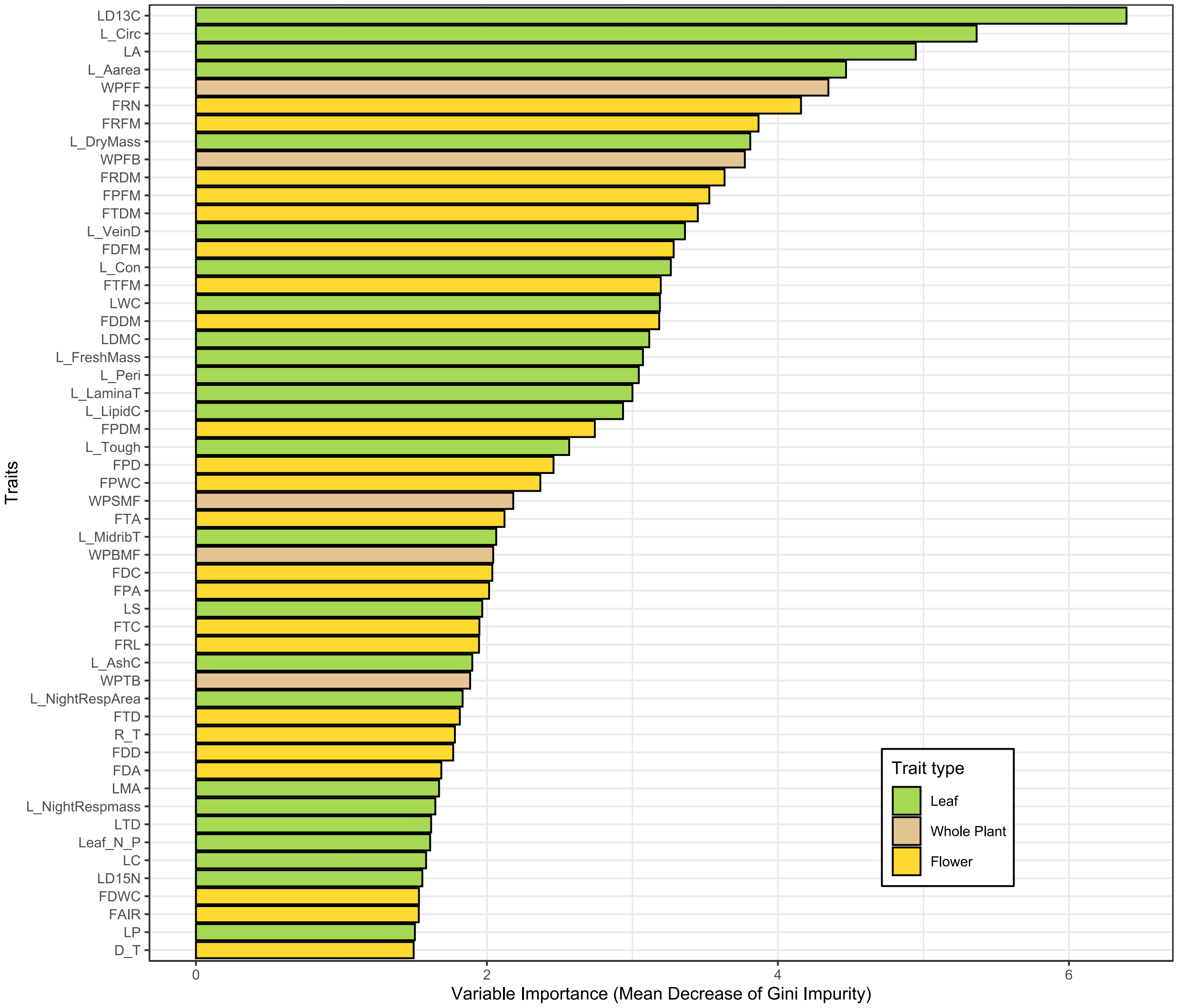

Figure S10. Optimal subset of divergence-relevant traits within the large perennial clade, ascertained by using a recursive feature elimination (RFE) method on the data set. The variable importance was calculated using the mean decrease of accuracy from a random forest classifier within the framework of RFE.

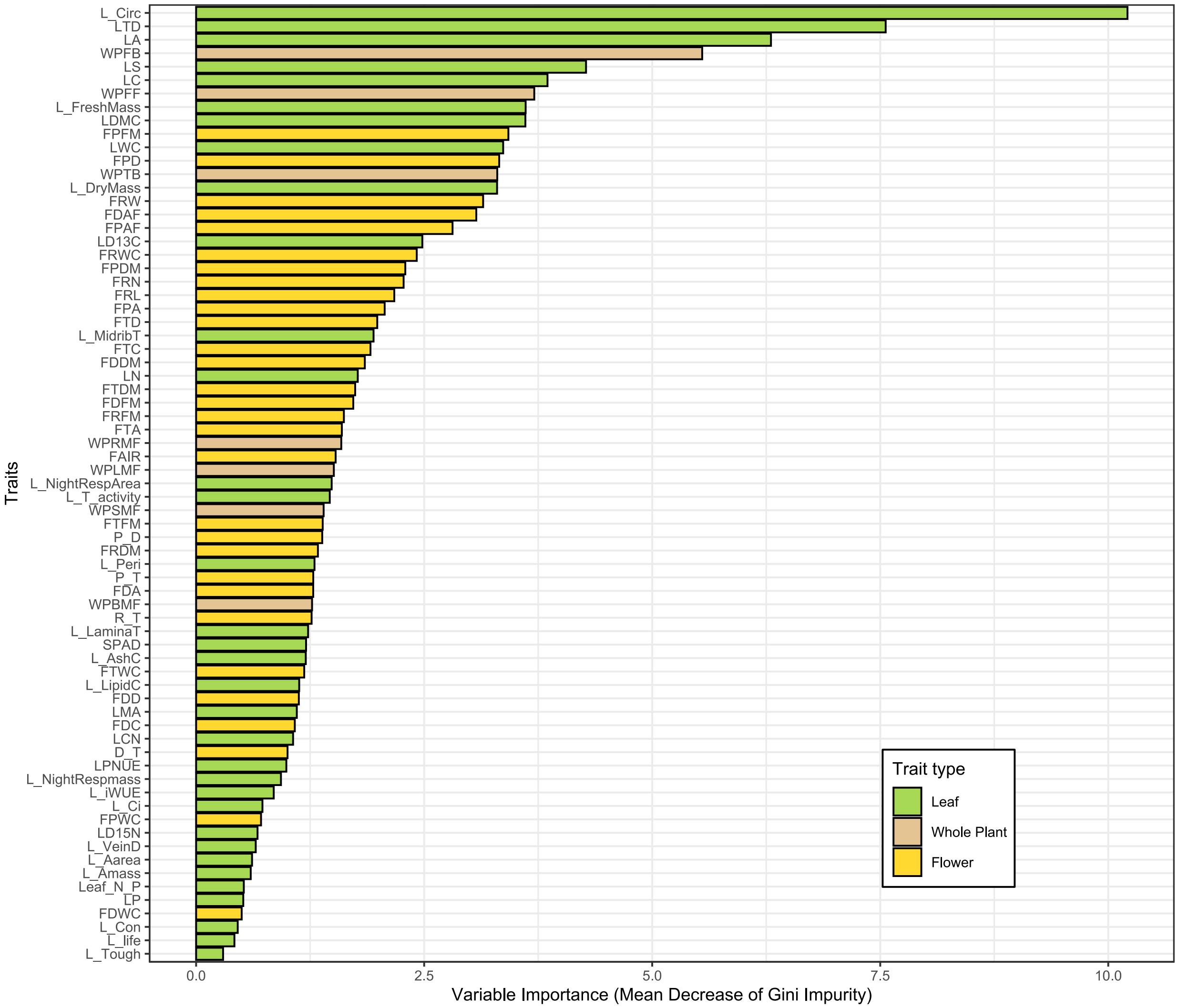

Figure S11. Relative importance of all 71 traits within the southeastern perennial clade, computed using Gini impurity by applying a random forest classifier to the training data. This was used to rank all the traits in the data set.

DOI 10.1002/aps3.11576

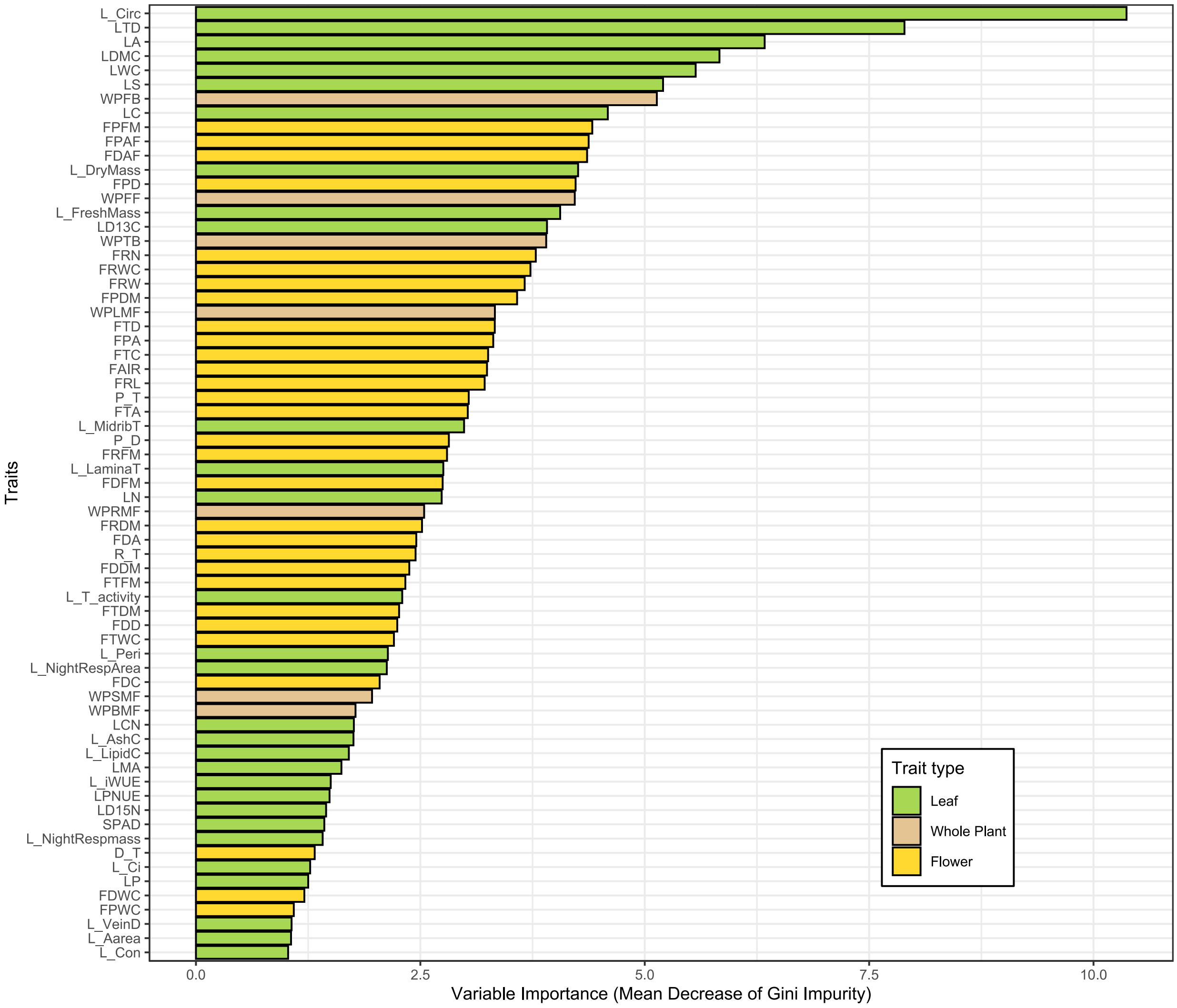

Figure S12. Optimal subset of divergence-relevant traits within the southeastern perennial clade, ascertained using a recursive feature elimination (RFE) method on the data set. The variable importance was calculated using mean decrease of accuracy from a random forest classifier within the framework of RFE.
